# Baricitinib with cyclosporine eliminates acute graft rejection in fully mismatched skin and heart transplant models

**DOI:** 10.3389/fimmu.2023.1264496

**Published:** 2023-09-06

**Authors:** Ramzi Abboud, Sena Kim, Karl Staser, Reyka G. Jayasinghe, Sora Lim, Parmeshwar Amatya, C. Corbin Frye, Benjamin Kopecky, Julie Ritchey, Feng Gao, Kory Lavine, Daniel Kreisel, John F. DiPersio, Jaebok Choi

**Affiliations:** ^1^ Division of Oncology, Section of Leukemia and Stem Cell Transplantation, Department of Medicine, Washington University School of Medicine, St. Louis, MO, United States; ^2^ Division of Dermatology, Department of Medicine, Washington University School of Medicine, St. Louis, MO, United States; ^3^ Division of Cardiothoracic Surgery, Department of Surgery, Washington University School of Medicine, St. Louis, MO, United States; ^4^ Division of Cardiology, Department of Medicine, Washington University School of Medicine, St. Louis, MO, United States; ^5^ Division of Public Health Sciences, Department of Surgery, Washington University School of Medicine, St. Louis, MO, United States

**Keywords:** baricitinib, JAK, Solid organ, graft, cyclosporine, immunosuppression

## Abstract

Solid organ transplant represents a potentially lifesaving procedure for patients suffering from end-stage heart, lung, liver, and kidney failure. However, rejection remains a significant source of morbidity and immunosuppressive medications have significant toxicities. Janus kinase (JAK) inhibitors are effective immunosuppressants in autoimmune diseases and graft versus host disease after allogeneic hematopoietic cell transplantation. Here we examine the role of JAK inhibition in preclinical fully major histocompatibility mismatched skin and heart allograft models. Baricitinib combined with cyclosporine A (CsA) preserved fully major histocompatibility mismatched skin grafts for the entirety of a 111-day experimental period. In baricitinib plus CsA treated mice, circulating CD4^+^T-bet^+^ T cells, CD8^+^T-bet^+^ T cells, and CD4^+^FOXP3^+^ regulatory T cells were reduced. Single cell RNA sequencing revealed a unique expression profile in immune cells in the skin of baricitinib plus CsA treated mice, including decreased inflammatory neutrophils and increased CCR2^-^ macrophages. In a fully major histocompatibility mismatched mismatched heart allograft model, baricitinib plus CsA prevented graft rejection for the entire 28-day treatment period compared with 9 days in controls. Our findings establish that the combination of baricitinib and CsA prevents rejection in allogeneic skin and heart graft models and supports the study of JAK inhibitors in human solid organ transplantation.

## Introduction

Solid organ transplant represents a potentially lifesaving procedure for patients suffering from heart, lung, liver, and kidney failure. However, rejection remains a significant source of mortality and morbidity in the short- and long-term through multiple mechanisms (1, 2). Human Leukocyte Antigen (HLA) -A, -B, and -DR matching can reduce the risk of rejection, but is not possible in all cases, especially for organs where the urgency of transplantation is high, the donor pool is small, and other donor/recipient factors (such as heart size) must be considered (1). Rejection is mediated through a variety of immunological pathways, which vary in incidence and severity between solid organ grafts. HLA disparity can result in both antibody and cellular based rejection. Hyperacute and early antibody-mediated rejection are caused by preformed HLA-specific antibodies, highlighting the importance of donor antibody screening prior to transplantation (3). Donor passenger and recipient antigen-presenting cells can play a role in instigating cellular rejection (4). The benefit of HLA matching varies across solid organ types (5). Viral infections, especially when affecting transplanted solid organs, have been implicated in rejection (6–9). Long-term pharmacologic immunosuppression is a cornerstone of solid organ transplantation. Immunosuppression regimens incorporate calcineurin inhibitors such as cyclosporine A (CsA), corticosteroids, antimetabolites such as mycophenolate mofetil, and cytotoxic immunosuppressants. These agents are associated with significant short- and long-term toxicities (10), including end organ renal and cardiovascular toxicity, increased risk of infection, and secondary malignancies. Furthermore, despite immunosuppression, rejection ultimately occurs in the majority of patients (4, 11, 12). Therefore, novel agents and immunosuppressive combinations are needed.

Janus kinases (JAK) and signal transducers and activation of transcription (STAT) were described in the early 1990s as a family of rapid membrane to nucleus signaling molecules that act downstream of over 50 cytokines (13). There are four members of the JAK family – JAK1, JAK2, JAK3, and TYK2 – and seven members of the STAT family – STAT1, STAT2, STAT3, STAT4, STAT5a, STAT5b, and STAT6 (14). These pathways are central to the normal function of the hematopoietic and immune systems. In general, loss of function mutations lead to immunodeficiency syndromes and gain of function mutations are associated with myeloproliferative syndromes (13). JAK inhibitors have been successfully employed in the treatment of graft-versus host disease (GVHD) after allogeneic hematopoietic cell transplantation, with ruxolitinib approved for steroid refractory acute and chronic GVHD, and several other JAK inhibitors under clinical investigation for prevention or treatment of GVHD (15, 16). JAK inhibitors can safely be given on a long-term basis evidenced by their use in the management of chronic rheumatologic and myeloproliferative diseases (17).

We sought to elucidate the role of JAK inhibitors in preventing solid organ rejection, alone and in combination with traditional immunosuppressants. The two JAK inhibitors used in this study are baricitinib and ruxolitinib (18, 19). Baricitinib is a JAK1/JAK2 inhibitor which is approved for the treatment of rheumatoid arthritis and under emergency use authorization for COVID19 and has been tested in other autoimmune diseases and for treatment and prevention of GVHD. Ruxolitinib is a JAK1/JAK2 inhibitor which is approved for the treatment of acute and chronic GVHD, myelofibrosis, and polycythemia vera. It has been tested in autoimmune diseases and GVHD prevention (**Table S1**) (20).

We hypothesized that JAK inhibition combined with traditional immunosuppression with CsA could prevent allograft rejection in fully major histocompatibility MHC-mismatched mouse skin and heart transplant models.

## Results

### Effect of JAK inhibitors on survival of mismatched allogeneic skin graft

We first examined the effect of JAK inhibitors on the survival of mismatched allogeneic skin grafts (**Materials and Methods, Figure S1**).

#### Baricitinib extends allogeneic skin graft survival in a MHC-mismatched skin graft model

**Figure 1:**
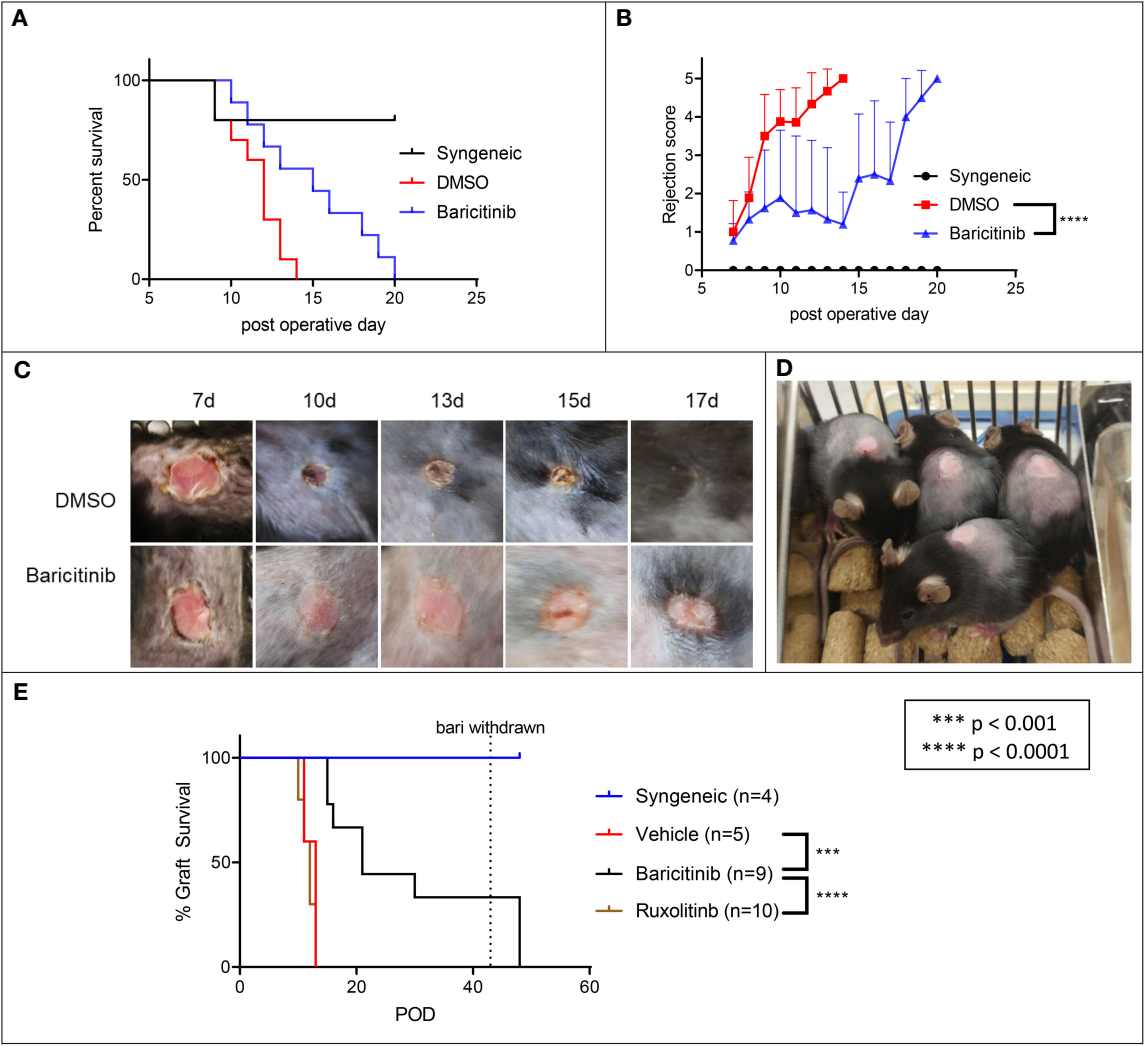
Baricitinib extends allogeneic skin draft survival in a MHC-Mismatched skin graft model, ruxolitinib does not. Dorsal ear skin was collected from BALB/c mice and grafted to the backs of fully MHC-mismatched B6 mice. Grafts were bandaged until POD 7 to protect grafts, then assessed daily for rejection. **(A)** Mean survival time (MST) of skin grafts was 12 days in rejection controls to 15 days in treated mice (p = 0.080). Syngeneic grafts were not rejected. **(B)** Rejection scores were lower in baricitnib treated mice compared with rejection controls (p < 0.0001). **(C)** Photographs taken throughout the post-grafting period reveal that differences in graft health became apparent visually at POD 10 and beyond. Grafts in rejection controls were smaller, less pink, and more crusty and necrotic appearing. **(D)** Skin grafts in baricitinib treated mice on POD 10 are maintained in size and remain viable. **(E)** We repeated the experiment, adding a ruxolitinib treatment arm. Treatment with ruxolitinib did not increase survival of allogeneic skin grafts. Baricitinib treatment again extended the survival of grafts (p < 0.0001).

We tested baricitinib alone in a fully MHC-mismatched BALB/c to B6 allogeneic skin grafting model. Mice were separated into three groups: syngeneic controls (n=5), rejection controls treated with 10% DMSO (n=10) and the baricitinib treatment group (n=10). Mice in the treatment groups were treated with baricitinib 400 μg or 10% DMSO subcutaneously (s.c.) daily from day -1 until graft rejection. Syngeneic control grafts were not rejected (**Figures 1A, B**). Compared with rejection controls, there was a trend towards longer graft survival (p = 0.080) and rejection scores were lower (p < 0.001) in the baricitinib group (**Figures 1A, B**). Differences in graft health became apparent visually at post-operative day (POD) 9 and beyond (**Figures 1C, D**). Mean graft survival time (MST) was longer in the baricitinib group compared with rejection controls, 12 days versus 15 days (**Figure 1A**) and mean rejection scores were significantly lower at all time-points after skin grafting in the baricitinib group compared with DMSO rejection control group. In the control and baricitinib groups, all skin grafts were ultimately rejected by day 14 and 20, respectively.

#### Frequencies of CD45^+^CD3^+^ cells are increased in allogeneic skin grafts of baricitinib treated mice in a MHC-mismatched model

We performed flow cytometry of allogeneic skin graft cells on POD 14 in our fully MHC-mismatched BALB/c to B6 allogeneic model. Mice were separated into three groups: syngeneic controls (n=3), rejection controls treated with 10% DMSO (N=4), and the baricitinib treatment group (n=3). At the time of harvest, syngeneic grafts were alive. Flow cytometry revealed 8.8% of live cells were CD45^+^ cells in the skin (**Figure S2A - top row, S2B**). Compared with syngeneic controls (8.8%), the frequencies of CD45^+^ cells were increased in both the baricitinib treated (20.6%) and rejection control (22.8%) skin grafts (**Figure S2A - middle and bottom rows, S2B**). There was a small increase in the percent CD45^+^CD3^+^ T cells in the allogeneic grafts treated with baricitinib (6.0%) and in the rejection controls (5.3%) compared with syngeneic controls (2.7%) (**Figure S2C**).

#### Ruxolitinib does not extend allogeneic skin graft survival in an MHC-mismatched model

We next tested ruxolitinib alongside baricitinib and rejection controls in a fully MHC-mismatched BALB/c to B6 allogeneic skin grafting model. Mice were separated into three groups: syngeneic controls (n=5), rejection controls treated with DMSO (n=5), the ruxolitinib treatment group (n=10) and the baricitinib treatment group (n=10). Mice in the treatment groups were treated with DMSO, ruxolitinib 400 μg s.c, or baricitinib 400 μg s.c. daily from day -1 until graft rejection or at most day 28. Syngeneic control grafts were not rejected (**Figure 1E**). Consistent with the previous experiment, graft survival was again significantly prolonged in the baricitinib treatment group with MST of 21 days (p < 0.0001, **Figure 1E**). However, time to rejection was not prolonged in the ruxolitinib treated group, MST 12 days, similar to the DMSO group (**Figure 1E**).

#### The combination of baricitinib and CsA indefinitely prevents allogeneic skin graft rejection in a MHC-mismatched model

**Figure 2:**
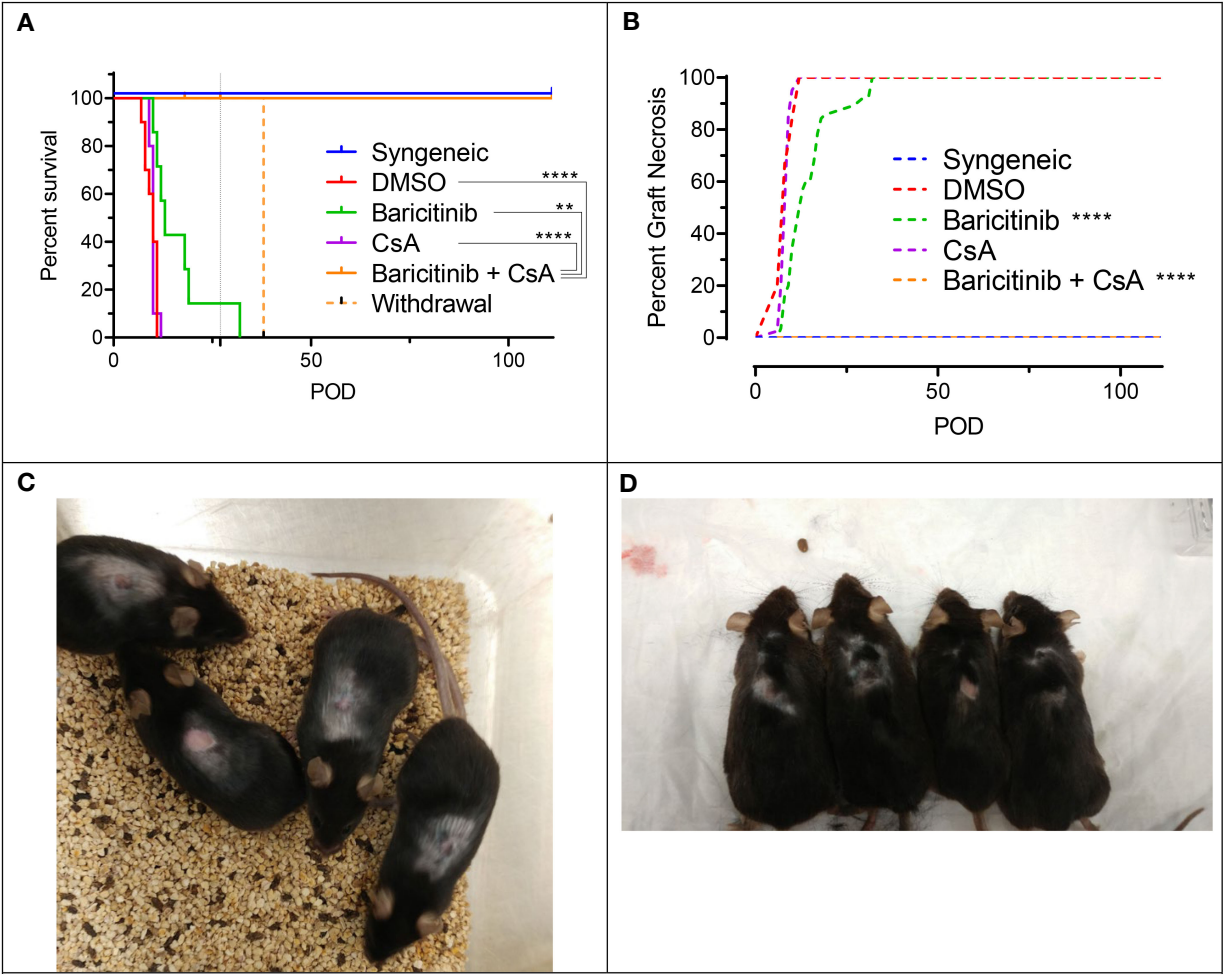
The combination of baricitinib and CsA indefinitely prevents allogeneic skin graft rejection in a MHC-mismatched Model. Dorsal ear skin was grafted from donors to recipients as previously described. Experimental groups included syngeneic controls, rejection controls, and baricitinib, CsA, and baricitinib plus CsA treatment groups. **(A)** Mean skin graft survival (MST) of experimental groups. Rejection was completely prevented in mice treated with the combination of baricitinib and CsA (vs. DMSO p < 0.0001, vs. CsA p < 0.0001, vs. baricitinib p = 0.010). In two mice, treatment withdrawal of baricitinib and CsA on POD 27 led to graft rejection on POD 38. The MST trended longer in the baricitinib treatment mice compared with CsA (p = 0.12) and rejection controls (p = 0.086), and CsA alone did not extend the survival of skin grafts (p=0.89). **(B)** Graft necrosis was lower in the baricitinib treatment group (vs. DMSO p < 0.0001), but completely absent in the combination baricitinib plus CsA treatment group (p < 0.0001). CsA alone did not reduce graft necrosis (p = 0.67). **(C)** Grafts in mice treated with baracitinib and CsA remain alive and healthy on POD 90 and **(D)** POD 111. **p < 0.01; ****p < 0.0001.

Given the important role of CsA in immunosuppression after organ transplantation, independent of JAK1/JAK2 inhibition, we next combined it with baricitinib in our fully MHC-mismatched BALB/c to B6 allogeneic skin transplantation model. Mice were separated into five groups: syngeneic controls (n=10), rejection controls treated with 10% DMSO (n=10), baricitinib alone treatment group (n=10), CsA alone treatment group (n=10), and baricitinib plus CsA treatment group (n=10). Treatment was given daily from day -1 until either day 111 or graft rejection (whichever came first), except for two mice in the baricitinib plus CsA group, where treatment was stopped on day 27 in the absence of rejection. Treatment dosing was as follows: baricitinib 400 μg s.c. daily, 10% DMSO by s.c. daily, and CsA 500 μg s.c. daily. The syngeneic controls experienced no graft rejection (**Figures 2A, B**). The 10% DMSO and CsA alone groups had similar early graft rejection, with MST of 11 days (**Figures 2A, B**). As previously shown, baricitinib alone led to a modest improvement in graft survival - MST 13 days (p = 0.086). However, mice treated with the combination of baricitinib and CsA experienced no graft rejection for the full 111-day duration of therapy (p < 0.0001) (**Figures 2A, B**). In the two mice where baricitinib and CsA were stopped on day 27, graft rejection occurred on day 38. The remainder of the mice in the baricitinib and CsA group continued to be treated and remained free of graft rejection through POD 111 (**Figures 2C, D**).

#### Baricitinib treatment suppresses CD4^+^, CD8^+^, and regulatory t cell subsets in peripheral blood and allogeneic skin grafts in MHC-mismatched model

**Figure 3:**
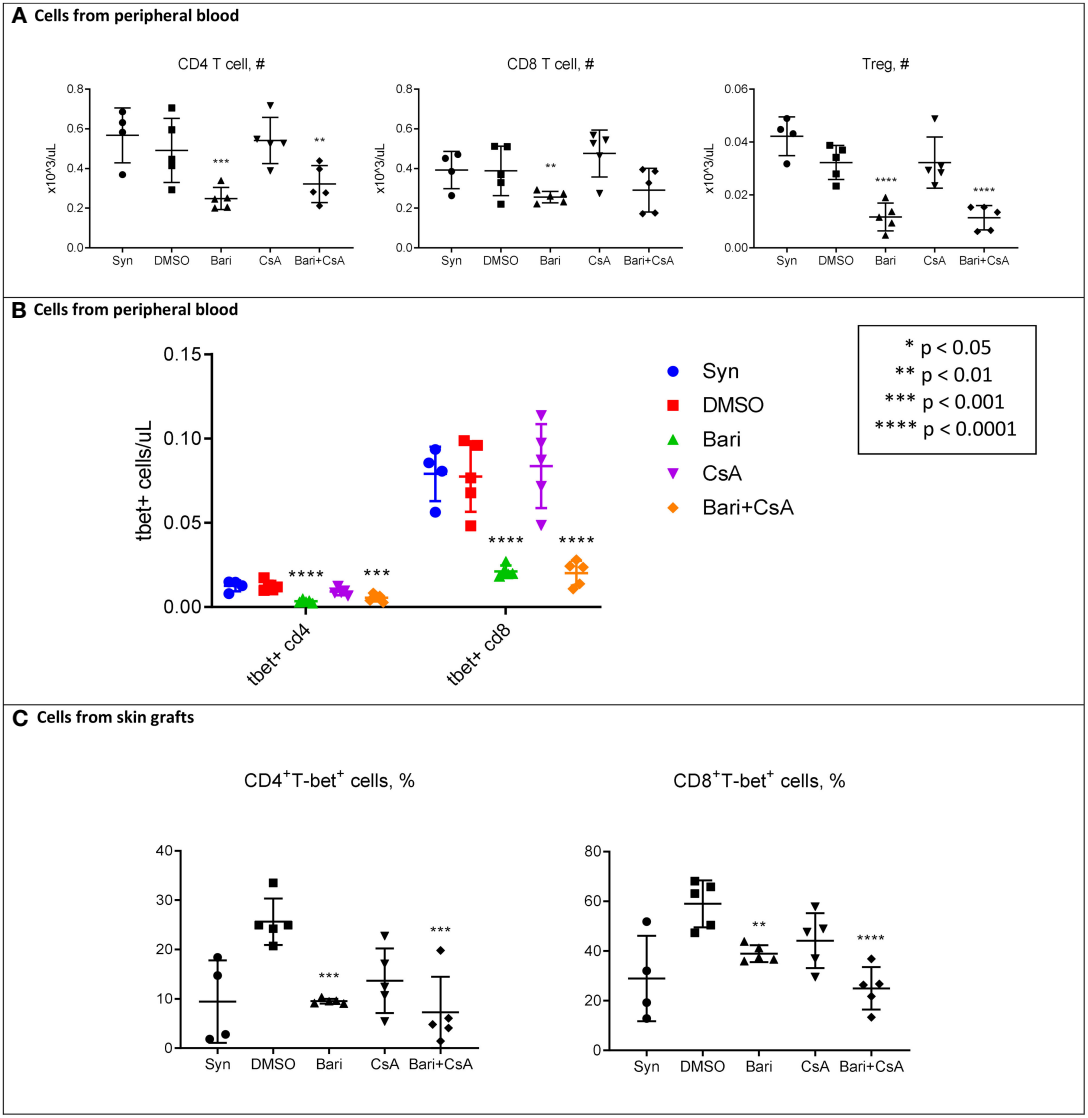
POD 5 flow cytometry of mononuclear cells from peripheral blood and skin in MHC-Mismatched Model. Cells were collected from peripheral blood or skin grafts and stained with fluorophore-conjugated antibodies and analyzed by flow cytometry as described in methods. Hematopoietic cells including T cells in both skin and peripheral blood were all (> 99%) recipient-derived (H-2Kb+) (**Supplemental Figure S8**). **(A)** Left: Peripheral blood circulating CD4^+^ T cells were reduced in both baricitinib (vs. syngeneic p = 0.0007, vs. DMSO p = 0.075, vs. CsA p = 0.0009, vs. baricitinib plus CsA p = 0.34) and baricitinib + CsA treated groups (vs. syngeneic p = 0.0058, vs. DMSO p = 0.035, vs. CsA p = 0.0083). Center: There was a similar trend for circulating CD8^+^ T cells, reduced in both baricitinib (vs. syngeneic p = 0.061, vs. DMSO p = 0.055, vs. CsA p = 0.0030, vs. baricitinib plus CsA p = 0.60) and baricitinib + CsA treated groups (vs. syngeneic p = 0.15, vs. DMSO p = 0.15, vs. CsA p = 0.0099). Right: Peripheral Blood circulating CD4^+^FOXP3^+^ regulatory T cells were decreased in both baricitinib (vs. syngeneic p < 0.0001, vs. DMSO p = 0.0001, vs. CsA p = 0.0001, vs. baricitinib plus CsA p = 0.95) and baricitinib + CsA treated groups (vs. syngeneic p < 0.0001, vs. DMSO p = 0.0001, vs. CsA p = 0.0001). **(B)** Peripheral blood CD4^+^T-bet^+^ and CD8^+^T-bet^+^ T cells are dramatically reduced in both baricitinib and baricitinib + CsA treated groups - Left CD4^+^T-bet^+^ baricitinib (vs. syngeneic p < 0.001, vs. DMSO p < 0.0001, vs. CsA p = 0.0013, vs. baricitinib plus CsA p = 0.17) and baricitinib + CsA treated groups (vs. syngeneic p = 0.0004, vs. DMSO p = 0.0002, vs. CsA p = 0.030) - Right. CD8^+^T-bet^+^ baricitinib (vs. syngeneic p < 0.0001, vs. DMSO p < 0.0001, vs. CsA p < 0.0001, vs. baricitinib plus CsA p = 0.92) and baricitinib + CsA treated groups (vs. syngeneic p < 0.0001, vs. DMSO p < 0.0001, vs. CsA p < 0.0001). **(C)** In mononuclear cells from skin grafts of both groups of baricitinib treated mice, CD4^+^T-bet^+^ T cells and CD8^+^T-bet^+^ T cells are decreased compared with rejection controls - Left. CD4^+^T-bet^+^ T cells baricitinib (vs. syngeneic p = 0.98, vs. DMSO p = 0.0004, vs. CsA p = 0.29, vs. baricitinib plus CsA p = 0.56) and baricitinib + CsA treated groups (vs. syngeneic p = 0.59, vs. DMSO p = 0.0001, vs. CsA p = 0.11) - Right. CD8^+^T-bet^+^ T cells baricitinib (vs. syngeneic p = 0.17, vs. DMSO p = 0.0070, vs. CsA p = 0.44, vs. baricitinib plus CsA p = 0.047) and baricitinib + CsA treated groups (vs. syngeneic p = 0.57, vs. DMSO p < 0.0001, vs. CsA p = 0.0091).

We performed flow cytometry on peripheral blood mononuclear cells (PBMCs) and mononuclear cells isolated from the skin grafts on POD 5. Circulating total white blood cells (WBCs), red blood cells (RBCs), and platelets were similar among groups (**Figure S4A**). Likewise, circulating myeloid cells, B cells, and overall T cells were not different among groups (**Figure S4A**). However, circulating CD4^+^ T cells and CD4^+^FOXP3^+^ regulatory T cell subsets were reduced in baricitinib treated groups, with a similar trend for CD8^+^ T cells (**Figure 3A**). We found that the number of CD8^+^T-bet^+^ T cells was dramatically decreased in both baricitinib groups (**Figure 3B**). In the skin grafts of both baricitinib treated groups, CD4^+^T-bet^+^ T cells and CD8^+^T-bet^+^ T cells are decreased compared with rejection controls (**Figure 3C**). Furthermore, the number of CD4^+^FOXP3^+^ regulatory T cells in the skin was lower in baricitinib treated groups compared with syngeneic controls (**Figure S5**). Baricitinib treatment had no effect on circulating effector, memory, or naïve subsets of CD4^+^ or CD8^+^ T cells (**Figure S4B**), and no effect on most immune subtypes found in skin grafts (**Figure S5**). Of note, virtually all hematopoietic cells in both the peripheral blood and the donor skin were CD45.1^+^ recipient derived cells, and a similar pattern was seen in the CD3^+^ subset (**Figure S6**).

#### Baricitinib plus CsA treatment Alters RNA expression in myeloid and lymphoid subsets

We performed single cell RNA (scRNA) sequencing on CD45^+^ cells harvested from skin grafts on POD 6. A total of 8,455 cells were captured across five experimental groups: syngeneic controls (n=5), rejection controls treated with 10% DMSO (n=5), baricitinib alone treatment group (n=5), CsA alone treatment group (n=5), and baricitinib plus CsA treatment group (n=5, **Table S2**).

**Figure 4:**
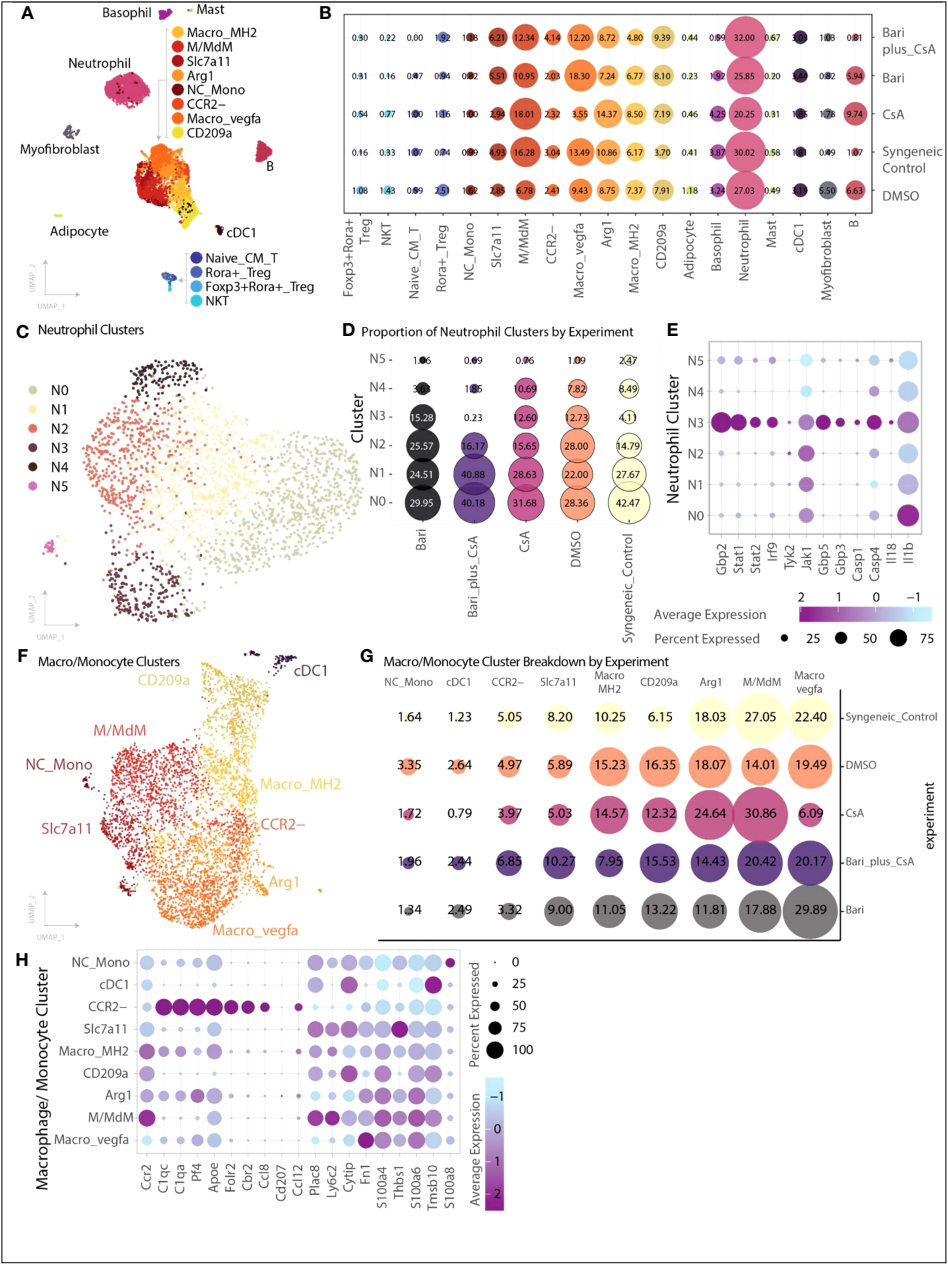
Single-cell transcriptional heterogeneity of immune subsets. Cells were collected from skin grafts on POD 6 and underwent CD45^+^ selection by positive selection magnetic-activated cell sorting. **(A)** Cell identification and clustering for all 8,455 cells analyzed across groups, cells are colored by cell type. The majority of cells were monocyte/macrophage, followed by neutrophils, with a smaller number of T cells, B cells, basophils, and other myeloid subtypes (Methods). **(B)** Proportion of cells identified for each cell type organized by experiment. Size of dot indicates proportion of cells, and color indicates cell type. **(C)** Six transcriptionally distinct populations of neutrophils were identified. **(D)** Relative proportion of each neutrophil cluster broken down by experimental group. The N3 cluster is dramatically reduced in the combination baricitinib plus CsA group (0.23%) and reduced in the syngeneic control group (4%) compared with the other three groups in which N3 neutrophils represent 12-15% of neutrophils. **(E)** Differentially expressed genes identified in neutrophil cluster N3 include genes associated with increased inflammasome activation signaling - IL-18, caspase-1, caspase-4, STAT1, STAT2, Guanylate Binding Protein 2, Guanylate Binding Protein 3, Guanylate Binding Protein 5, Interferon 9. Size of dot indicates percent of cells expressing the gene of interest while color indicates average expression. **(F)** Nine transcriptionally distinct populations of monocyte and macrophage related cells were identified. Each dot is colored by cell type. **(G)** Proportion of cells for each monocyte/macrophage subset by experimental group. Size of dot indicates proportion while color indicates experimental group. The CCR2^-^ population is increased in the baricitinib plus CsA group compared with the other single agent treatment groups (baricitinib plus CsA 6.85%, CsA 3.97%, baricitinib 3.32%). **(H)** Differentially expressed genes across each of the macrophage subsets highlighting CCR2^+^ and CCR2- subsets.

Across each of the samples the largest population of cells identified was neutrophils, followed by several populations of myeloid cells, and smaller subsets of T cells and other immune subsets (**Figures 4A, B**). B cells made up a very small proportion of cells in the syngeneic control (1.07%) and combined treatment (0.81%) groups.

We identified six populations of neutrophils and found that neutrophil cluster 3 (N3) was dramatically lower in both the baricitinib plus CsA (0.23%) and syngeneic control (4%) groups relative to both single agent groups (baricitinib:15%; CsA:13%) or DMSO control (13%) (**Figures 4C, D**). Cluster N3 exhibited increased inflammasome activation signaling (IL-18, Casp1, Casp4, Stat1, Stat2, Gbp2, Gbp3, Gbp5, Irf9, **Figure 4E**) (21). Gene set enrichment analysis of top differentially expressed genes (>1.0 average log2FC) from N3 suggests hallmark gene sets including interferon gamma response, genes upregulated by IL6 signaling and genes up regulated during transplant rejection are enriched in this neutrophil cluster (Methods).

Macrophage and monocyte subsets are represented by nine transcriptional populations (**Figure 4F**). In our skin grafts, we identified a cluster of CCR2^-^ macrophages (MC CCR2^-^), which mirrors the expression profile of recently described CCR2^-^ macrophages in a murine cardiac allograft model associated with extended cardiac allograft survival and reduced immunologic rejection (22). This MC CCR2^-^ population is nearly doubled in size in the baricitinib plus CsA group relative to other single agent treatment groups (baricitinib plus CsA 6.85%, CsA 3.97%, baricitinib 3.32%, **Figures 4B, G**). The remaining MCs exhibit gene expression consistent with CCR2^+^ macrophages (**Figure 4H**).

### Effect of baricitinib plus CsA on survival of mismatched allogeneic heart graft

Given the clinical need for novel immunosuppression strategies in heart transplantation, we next examined the effect of baricitinib plus CsA on the survival of HLA-mismatched allogeneic heart grafts.

#### The combination of baricitinib and CsA prevents allogeneic heart graft rejection

**Figure 5:**
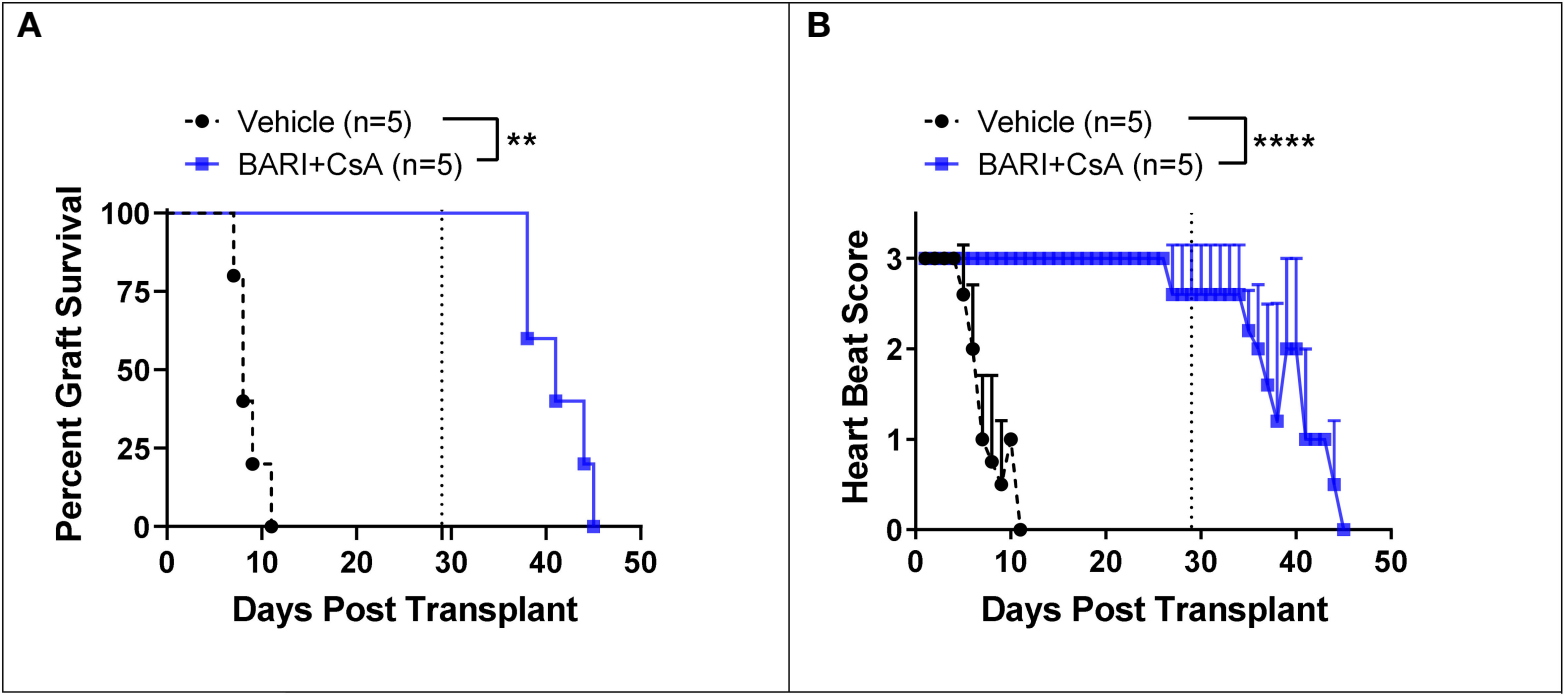
Mismatched heart grafts from BALB/c donors to B6 recipients. **(A)** In the baricitinib plus CsA treatment group, all donor hearts survived throughout the 28 day treatment period, while untreated controls were all rejected by POD 11 (p = 0.0018). After treatment withdrawal on day 29, MST of hearts was 14 days and all hearts were rejected by 17 days after treatment withdrawal. **(B)** Heart beat scores were maintained in the baricitinib plus CsA group throughout the 28 day treatment period, then began to fall seven days after treatment withdrawal (p < 0.0001). **p < 0.01; ****p < 0.0001.

We tested the combination of baricitinib and CsA in a fully MHC-mismatched BALB/c to B6 allogeneic heart transplantation model. Mice were separated into two groups: untreated rejection controls (n=5), and a baricitinib plus CsA treatment group (n=5). Treatment was given daily from day 0 until POD 28. Treatment dosing was as follows: baricitinib 400 μg s.c. injection and CsA 500 μg s.c. injection. In rejection controls, time to rejection was consistent with previously published results (23–25), MST for hearts was 9 days and all hearts were rejected by day 11 (**Figures 5A, B**). In contrast, in the baricitinib plus CsA group, no hearts were rejected, and heart beat scores were maintained during the treatment period of 28 days. After treatment was withdrawn on day 28, MST of hearts was 14 days, and all hearts were rejected by 17 days after withdrawal of treatment with both baricitinib and CSA (**Figures 5A, B**). Due to resource limitations, we were unable to include a CsA alone treatment group, and must draw on published literature. A study with similar design, using intraperitoneal injections of CsA at several doses including 20 mg/kg/day (approximately 400 μg daily) and 30 mg/kg/day (approxlimately 600 μg daily), demonstrated heart graft survival was 89% and 50% respectively at 30 days (26). Heart beat scores were not maintained during the treatment period, and had fallen to 1.3 and 1.5 respectively (26).

#### Effect of baricitinib and CsA treatment on peripheral blood CD4^+^, CD8^+^, and regulatory t cell subsets in heart allograft model

We performed flow cytometry on peripheral blood on POD 6 in all mice and on POD 35, seven days after treatment discontinuation, in baricitinib plus CsA mice. While in our skin transplant model at POD 5 CD4^+^FOXP3^+^ regulatory T cells in peripheral blood were reduced, in our heart transplant model at POD 6 the reduction in CD8^+^FOXP3^+^ T cells in peripheral blood was more prominent (**Figures S8A, B**). At the later time point, POD 35, both CD4^+^FOXP3^+^ and CD8^+^FOXP3^+^ regulatory T cells had increased. Consistent with the skin transplant model, CD8^+^T-bet^+^ T cells in peripheral blood were significantly reduced (**Figures S8C, D**). Of note, seven days after the last dose of baricitinib plus CsA, the percent CD8^+^T-bet^+^ T cells was significantly increased and comparable to vehicle control at day 6 after transplantation in the baricitinib plus CsA group. RORγt^+^ T cells, Th17 helper T cells important in inflammatory processes and allograft rejection, were not different between treatment groups (**Figures S8E-H**). Proportions of T cell subsets were not different between groups (**Figure S7**).

#### Effect of baricitinib and CsA treatment on cell subsets infiltrating heart allograft using immunofluorescence

**Figure 6:**
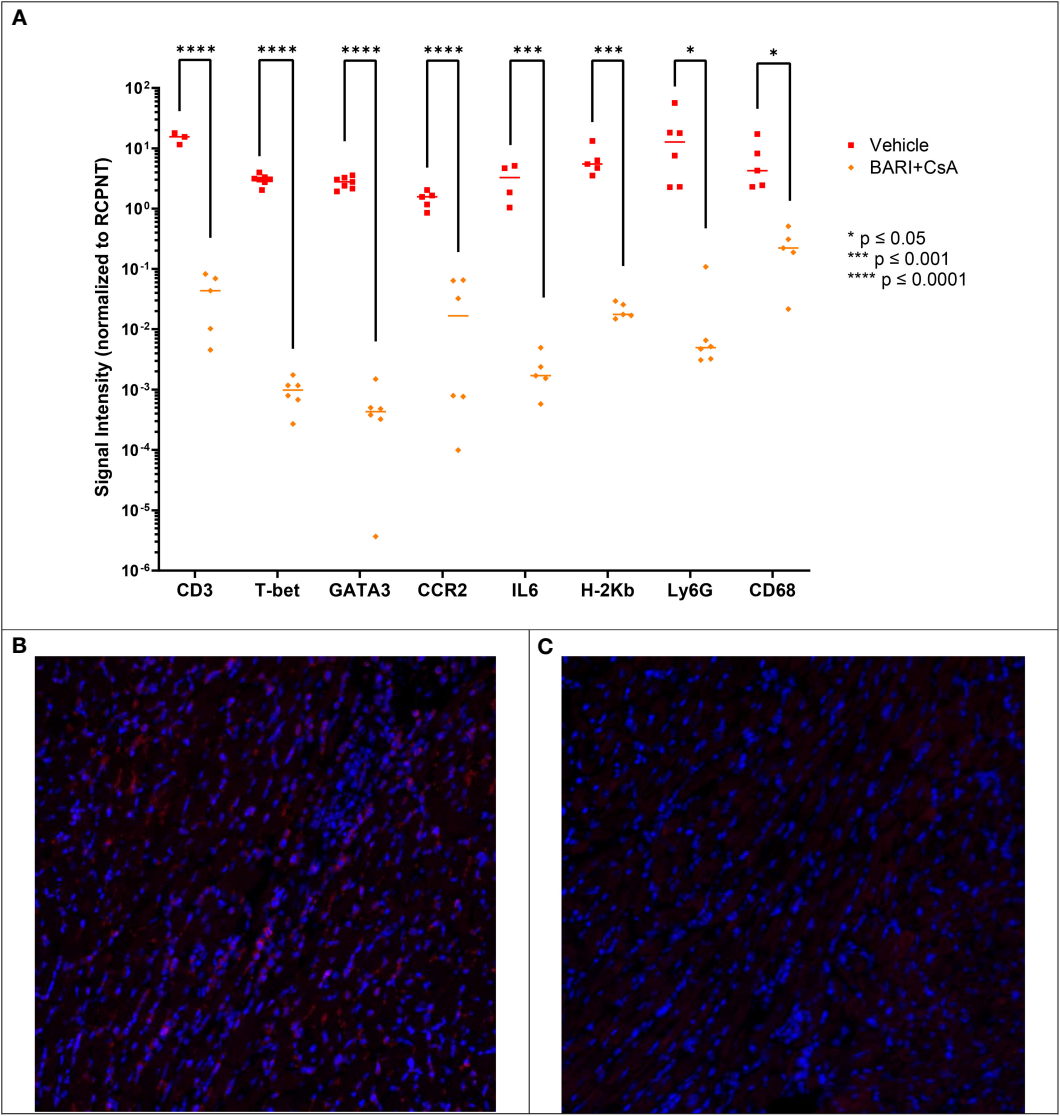
Mismatched heart grafts from BALB/c donors to B6 recipients, IF performed on FFPE heart tissue harvested on POD 7. **(A)** Measured CD3, T-bet, GATA3, CCR2, IL6, H-2Kb, Ly6G, and CD68 were lower in the Bari + CsA group compared with vehicle controls. Representative IF images of CD3 staining in vehicle **(B)** and baricitinib plus CsA treated **(C)** groups, where red represents positive CD3 staining. *p < 0.05; ***p < 0.001; ****p < 0.0001.

We repeated the heart graft experiments described above in order to perform immunofluorescence (IF) microscopy on formalin-fixed paraffin-embedded (FFPE) heart tissue harvested on POD 7. We included two groups: vehicle rejection controls (n=2) and baricitinib plus CsA (n=4). We found significantly lower (100-1000 fold) levels of CD3^+^, T-bet^+^, GATA3^+^, CCR2^+^, IL-6^+^, host H-2K^b^, and myeloid markers Ly6G and CD68 (**Figure 6A**) immunostaining in the baricitinib plus CsA treatment group compared with the rejection control group.

## Discussion

To the best of our knowledge, this is the first study demonstrating that the combination of JAK inhibition and CsA prevents fully MHC-mismatched skin and cardiac allograft rejection for the entirety of an extended therapy period. Likewise, we showed that combined therapy maintained heart graft survival and function until withdrawal of treatment. We analyzed the immune profile and transcriptional signature in circulating blood and grafts using flow cytometry, IF, and single cell RNA sequencing to further explore the clinical differences in graft survival.

Among JAK inhibitors, when used as single agents, baricitinib alone extended the survival of mismatched skin grafts while ruxolitinib did not. Baricitinib uniquely combines JAK1 and JAK2 best-in-class inhibition with selectivity, relatively sparing JAK3 and TYK2 (20). In addition, our previous work has demonstrated that baricitinib was superior to ruxolitinib and the best-in-class JAK1/JAK2 inhibitor for both the treatment and prevention of graft vs. host disease in mouse MHC-mismatched allogeneic stem cell transplant models (27–29). There are a number of differences between the two JAK inhibitors, including baricitinib is renally excreted and ruxolitinib is not, baricitinib is CYP-450 independent and ruxolitinib is not, baricitinib blocks the PKN1 kinase, involved in T cell trafficking, and ruxolitinib does not (30). We next demonstrated that the combination of baricitinib and CsA prevents rejection for an extended treatment period (the entire treatment duration). When treatment was withdrawn early on POD 27, graft rejection occurred within 11 days suggesting ongoing immunosuppression as opposed to inducing a state of tolerance or anergy.

We next tested baricitinib and CsA in a mismatched allogeneic heart graft model. We found that heart grafts could be maintained for the duration of a 28-day treatment period, with preservation of heart beat score. Heart grafts were rejected at a median of 14 days after withdrawal of treatment. Like the skin grafting experiments, these data suggest that the combination of baricitinib plus CSA did not induce a state of tolerance or anergy since both skin grafts and cardiac allografts in mice treated with baricitinib and CSA were rejected within 2-3 weeks of discontinuing both agents. Due to resource limitations during the pandemic, we were unfortunately unable to test a longer treatment period in our heart transplant model. However, it is possible that, consistent with our skin graft model, a longer treatment period may help prevent or delay rejection after withdrawal of therapy.

Interestingly, characterization of T cells revealed that in both the skin grafts and peripheral blood of baricitinib treated groups, T-bet^+^ T cells were significantly decreased compared with rejection controls. Similarly, in our heart transplant model T-bet^+^ T cells were reduced in the heart allograft (by IF) of baricitinib plus CsA treated mice compared to rejection control mice. Baricitinib has previously been shown to reduce the expression of T-bet in T cells; it is downstream of JAK1/2 and upsteam of CXCR3 (27–29). Disruption of T-bet signaling skews T cell differentiation away from a Th1 phenotype and towards Th2 and regulatory phenotypes, which reduces GVHD in preclinical bone marrow transplantation models (31). The reduction in T-bet^+^ T cells, mediated by baricitinib, may be necessary but not sufficient to protect against graft rejection, given that baricitinib alone did not prevent skin allograft rejection. Contrary to previous studies, in both our skin graft and heart transplant model, reduction in T-bet^+^ T cells was not associated with increased FOXP3^+^ regulatory T cells. In fact, the number of FOXP3^+^ regulatory T cells was lower in grafts and in circulation in our skin allograft model. Previous work has highlighted the role of CD4^+^FOXP3^+^ regulatory T cells in preventing rejection and inducing tolerance in transplantation (32, 33). Adoptive therapy including regulatory T cells has been shown to prevent rejection in a mouse heart allograft model, establishing the role of these cells in organ tolerance (34). This suggests that JAK inhibition with baricitinib may preserve solid organ grafts independent of regulatory T cells, and may explain why tolerance was not achieved in these models.

Single cell RNA sequencing revealed differences in the neutrophil and macrophage/monocyte compartments in baricitinib treated groups. A subset of inflammatory neutrophils, N3, was dramatically lower in the baricitinib plus CsA treatment group as well as the syngeneic control. Top differentially expressed genes in N3 include caspase-1, caspase-4, IL-18, STAT1, and STAT2, which are all associated with the NOD, LRR, and pyrin domain-containing 3 (NLRP3) protein inflammasome (35, 36). Neutrophils, as the most abundant cell in the leukocyte compartment, play an important part of mediating innate inflammation, activation of the NLRP3 inflammasome, and neutrophil extracellular traps (NETs) (36, 37). NLRP3 inflammasome activation and NETs have been associated with a wide range of pathology, including thrombosis, ischemia reperfusion injury, severe COVID-19, acute GVHD, and solid organ graft rejection (36, 38). In skin allograft models, the P2X7-NLRP3 activation pathway is associated with higher IL-18 production and secretion and a TH1/IFNγ alloimmune response resulting in graft injury (39). In heart allograft models, NLRP3 inhibition suppresses caspase-1 activity and improves perfusion pressures (40). JAK inhibition has been demonstrated to decrease NLRP3 inflammasome expression through reduction in STAT3 phosphorylation, leading to acetylation of the NLRP3 promoter (41, 42). Therefore, in the baricitinib plus CsA treatment groups, the observed dramatic increase in skin and heart graft survival may be partially mediated through JAK1/2 inhibition, reduction in STAT3 phosphorylation, and acetylation of the NLRP3 promoter.

The CCR2^-^ macrophage cluster was nearly doubled in size in skin grafts of baricitinib plus CsA treated mice. Recent work suggests that CCR2^-^ macrophages have a protective function through masking proinflammatory signaling from donor CCR2^+^ macrophages, and that recipient T cells may play an active role in eliminating donor CCR2^-^ macrophages (22). These immune cells do not work in isolation, and the effect of JAK inhibition on neutrophils and monocytes is likely interrelated. This is supported by the observation that CCR2^+^ macrophages play a role in neutrophil recruitment in heart grafts and are associated with immunologic rejection compared to CCR2-/- monocytes whose relative abundance is associated with prolonged graft survival and reduced immunologic rejection (43).

Our study is not without limitations. We were resource limited in both the number of and the duration of our heart transplant experiments. As a result, single agent CsA and baricitinib comparison groups were not included. The duration of therapy in our heart transplant model may not have been optimal to highlight the extended benefit of baricitinib plus CsA therapy, as we saw no rejection until treatment was withdrawn. In our skin transplant model, we observed rejection after treatment withdrawl even with very long treatment periods. We were unable to test very long treatment periods in the heart transplant model.

Our findings establish the combination of baricitinib and CsA as an immunosuppressive regimen with the potential to indefinitely prevent rejection in mouse skin and cardiac allogeneic solid organ transplant models. We identified several pathways through which rejection may be mitigated, including reduction in T-bet^+^ T cells, inflammatory neutrophils, and increase in CCR2^-^ macrophages. The incorporation of baricitinib, a relatively well tolerated drug for which there is extensive clinical experience as a chronic medication, may allow for reduction in dose of more toxic immunosuppressive agents including calcineurin inhibitors. Ruxolitinib, a balanced JAK1/JAK2 inhibitor with similar specificity to baricitinib has been approved for the treatment of steroid refractory acute GVHD and chronic GVHD. Other JAK inhibitors such as itacitinib (JAK1 specific) have been used to prevent GVHD when used as prophylaxis for haploidentical allogeneic stem cell transplant (44), for CRS mitigation induced by CAR T therapy (45) and for multiple other inflammatory disorders (17).

To our knowledge this is the first report of JAK1/JAK2 inhibitors and specifically baricitinib being used to prevent or reduce allograft rejection after tissue or solid organ transplantation. The combination of tofacitinib, a JAK3 inhibitor, and CTLA4-Ig has been demonstrated to induce long term allograft survival through inhibition of dendritic and T cells, with tofacitinib restoring the effect of CTLA4-Ig in the inflammatory setting (46). These data support our own data suggesting that JAK inhibition alone may be insufficient to prevent rejection, but combinations can lead to long term allograft survival. There has been only a single first-in-human clinical trial testing a JAK inhibitor for prevention of allogeneic solid organ rejection – tofacitinib after renal transplantation (47). In this study patients received standard immunosuppression in addition to either CsA or tofacitinib (at two dose levels). Patients receiving tofacitinib had improved renal function and less chronic allograft histological injury, but the same incidence of acute rejection. However, tofacitinib was associated with a higher rate of serious infections, cytopenias, and post-transplant lymphoproliferative disorders, and further studies have not been conducted. These complications were associated with high exposure levels, and there is interest in studying tofacitinib with patient adjusted dosing (48, 49). JAK1/2 inhibitors have been given to patients with myeloproliferative diseases for many years without significant infectious complications.

Our work supports the further preclinical and clinical exploration of JAK1/JAK2 inhibitors such as baricitinib plus other immunosuppressive agents such as CsA, as prophylaxis in human allogeneic solid organ transplantation.

## Materials and methods

### Mice

C57BL/6 (B6; H-2^b^) and BALB/c (H-2^d^) and mice were obtained from The Jackson Laboratory. Animal care and euthanasia were approved by the Washington University School of Medicine Animal Studies Committee. Six- to 12-week-old mice were used.

### Skin graft transplantation model

Dorsal ear skin was harvested from donor mice on ice and grafted to the back of recipient mice. Grafted skin was bandaged postoperative days (POD) 0 through 7, then assessed daily for rejection (**Figure S1**). In syngeneic controls, B6 mice served as donors and recipients. In rejection controls and treatment groups BALB/c (MHC-major mismatch) mice served as donors and B6 mice served as recipients. Graft rejection was scored from 0 – Intact Graft through 5 – Complete rejection (>95%) (50). Surgical failure was seen in less than 15% of procedures.

### Heart transplantation model

Cardiac grafts were harvested from BALB/c mice and were transplanted heterotopically into the abdomen of B6 recipients. After appropriate anesthesia and sterilization, donor surgery was performed through a median laparosternotomy with injection of intravenous heparin. The heart graft was harvested and stored in cold saline. For the recipient surgery, an appropriate plane of anesthesia was established, the surgical site was sterilized, and the surgery was performed through a laparotomy incision. Two surgical anastomoses were made between the donor graft ascending aorta and pulmonary artery to the recipient intra-abdominal aorta and inferior vena cava, respectively. Grafts resumed a regular heartbeat immediately following reperfusion. Postoperatively, mice were monitored closely for signs of distress and the graft function was assessed by daily palpation of the heart graft in the mouse’s abdomen. Heart graft function was assessed using the following heart beat score scale: 3 - soft graft with strong contraction, 2 - mild turgor and mild contraction, 1 - hard turgor and weak contraction, 0 - no palpable contraction, as scored by two independent observers and averaged (51).

### Administration of treatments

Baricitinib 400 μg was injected s.c. daily starting on day -1. Ruxolitinib 400 μg was injected s.c. daily starting on day -1. Cyclosporine A (CsA) was given s.c. 500 μg daily.

### Statistics

The determination of sample size and data analysis for this study followed the general guideline for animal studies (52). Skin and heart graft survival were described using Kaplan-Meier product limit method and compared by log-rank test, followed by *post-hoc* multiple comparisons for between-group differences of interest. All other data were summarized using means ± standard errors and the between-group differences were compared by two-sample t-test, one-way ANOVA, or linear mixed model for repeated measurement data, as appropriate. The normality of data was assessed graphically based on residuals and similarity of variance across groups was also assessed visually by checking the estimated variance of each group. A logarithm transformation was performed as necessary to better satisfy the normality and homoscedasticity assumptions. The resultant p-values were adjusted by Tukey’s test for multiple comparisons. Based on the law of diminishing returns, Mead recommended that a degree of freedom (DF) of 10-20 associated with error term in an ANOVA will be adequate for a pilot study to estimate preliminary information (53). All analyses were two-sided and significance was set at a p-value of 0.05. The statistical analyses were performed using SAS 9.4 (SAS Institutes, Cary, NC).

## Data availability statement

The original contributions presented in the study are included in the article/**Supplementary Material**, further inquiries can be directed to the corresponding author.

## Ethics statement

No human studies are presented in the manuscript. The animal studies were approved by Animal Studies committee at Washington University. The studies were conducted in accordance with the local legislation and institutional requirements. Written informed consent was not obtained from the owners for the participation of their animals in this study because Animal Studies committee at Washington University. No potentially identifiable images or data are presented in this study.

## Author contributions

RA: Conceptualization, Data curation, Formal Analysis, Funding acquisition, Investigation, Methodology, Software, Visualization, Writing – original draft, Writing – review & editing. SK: Methodology, Visualization, Writing – review & editing. KS: Conceptualization, Funding acquisition, Investigation, Methodology, Visualization, Writing – review & editing, Formal Analysis. RJ: Methodology, Writing – review & editing, Formal Analysis, Investigation. SL: Methodology, Visualization, Writing – review & editing, Formal Analysis, Investigation. PA: Investigation, Visualization, Writing – review & editing, Formal Analysis, Methodology. CF: Methodology, Writing – review & editing. BK: Investigation, Methodology, Writing – review & editing. JR: Methodology, Writing – review & editing. FG: Formal Analysis, Investigation, Writing – review & editing. KL: Conceptualization, Supervision, Writing – review & editing, Formal Analysis, Investigation. DK: Conceptualization, Supervision, Writing – review & editing, Formal Analysis, Investigation. JD: Conceptualization, Funding acquisition, Investigation, Project administration, Supervision, Writing – original draft, Writing – review & editing, Formal Analysis. JC: Conceptualization, Funding acquisition, Investigation, Methodology, Project administration, Supervision, Visualization, Writing – review & editing, Formal Analysis.
