# Supplementary material for: Baricitinib with cyclosporine eliminates acute graft rejection in fully mismatched skin and heart transplant models
Source: Front Immunol. 2023 Sep 6;14:1264496. doi: 10.3389/fimmu.2023.1264496 (PMC10511772; doi:10.3389/fimmu.2023.1264496)
Supplement: Supplementary file 1 [file DataSheet_1.docx]

Supplementary Materials for

**Baricitinib with Cyclosporine Eliminates Acute Graft Rejection in Fully Mismatched Skin and Heart Transplant Models**

Ramzi Abboud *et al.*

Corresponding authors: Ramzi Abboud, MD. Email: rabboud@wustl.edu

John F. Dipersio, MD, PhD. Email: jdipersi@wustl.edu

Jaebok Choi, PhD. Email: jchoi25@wustl.edu

**The PDF file includes:**

Materials and Methods:

Flow cytometry

Single Cell RNA Sequencing

Immunofluorescence microscopy on Formalin-Fixed

Paraffin-Embedded Tissues

Immunofluorescence protocol,

Supplementary Method 1, Gating strategy for T cells and FOXP3+ Subsets in Blood

Supplementary Method 2, Gating strategy for CD45+ Hematopoietic Cells and T cells in Skin

Supplementary Method 3, Gating strategy for T-bet+ T cells

Supplementary Method 4, Gating strategy for T cell Subsets in Heart

Supplementary Method 5, Gating strategy for T cell FOXP3, GATA3, T-bet, and RORγt in Heart

Figures S1 to S8

Tables S1 to S5

**SUPPLEMENTARY MATERIALS**

**Supplemental Methods:**

**Flow Cytometry**

Mouse blood and skin were collected from the recipients. Skin was mashed with syringe plunger and the mash was passed through the 70 μm cell strainer to make single cell suspension. Red blood cells in single cell suspension and blood (50 μl of whole blood per sample) were removed by ACK lysis buffer. 1×10^6^ cells from the single cell suspension or 50 μl of blood were stained with fluorochrome-conjugated antibodies after blocking with rat serum. For the intracellular staining, cells were fixed with FOXP3 transcription factor fixation buffer for 30 min at room temperature after surface staining. Intracellular nuclear protein staining (FOXP3, T-BET, GATA3, and RORγt) was prepared using the FOXP3/Transcription Factor Staining Buffer Set according to the manufacturer’s instructions (eBioscience, protocol B for intracellular protein). The antibodies used for flow cytometric analyses for mouse cells in the skin model **(Table S3)** and heart model **(Table S3)** are included in the supplemental materials. All cells were analyzed on Attune® NxT (Thermo Fisher Scientific, Waltham, MA). All data collected from flow cytometry were analyzed with the Flow Jo software (FlowJo LLC, Ashland, OR).

**Single Cell RNA Sequencing**

Cell collection

Skin was mashed with syringe plunger and the mash was passed through the 70 μm cell strainer to make single cell suspension. Red blood cells in single cell suspension were removed by ACK lysis buffer. Cells were washed and incubated with anti-mouse CD45 BV421 (BD biosciences 563890) antibodies per technical data sheet. CD45^+^ cells were isolated by flow cytometry using the Sony Synergy five laser, 22 color sorter. Cells were then counted at 40,000 viable cells were provided for single cell RNA sequencing.

Single Cell Library Prep and Sequencing

Utilizing the Chromium Next GEM Single Cell 3’ GEM, Library & Gel Bead Kit v3.1 and Chromium instrument, approximately 17,500 to 25,000 cells were partitioned into droplets to achieve single-cell resolution for a maximum of 10,000 to 15,000 individual cells per sample (10x Genomics, 1000269). The resulting cDNA was tagged with a common 16nt cell barcode and 10nt Unique Molecular Identifier during the RT reaction. Full-length cDNA from poly-A mRNA transcripts was enzymatically fragmented and size-selected to optimize the cDNA amplicon size (approximately 400bp) for library construction (10x Genomics).The concentration of the 10x single-cell library was accurately determined through qPCR (Kapa Biosystems) to produce cluster counts appropriate for the HiSeq 4000 or NovaSeq 6000 platform (Illumina). 26x98bp sequence data were generated targeting 50K read pairs/cell, which provided digital gene expression profiles for each individual cell.

Single Cell Data Preprocessing

For each sample, we obtained the unfiltered feature-barcode matrix per sample by passing the demultiplexed FASTQs to Cell Ranger v6.1.1 ‘count’ command using default parameters and the prebuilt refdata-gex-mm10-2020-A for single cell RNA (scRNA). Seurat v3.1.2 *(55, 56)* was used for all subsequent analyses. First, a series of quality filters was applied to the data to remove those barcodes which fell into any one of these categories recommended by Seurat: total transcript counts (< 300); genes expressed (< 200 & > 10,000) and UMIs (< 1,000 & > 10,000); percent of mitrochondrial counts (> 10%). Each sample was scaled and normalized using Seurat’s ‘SCTransform’ function to correct for batch effects (with parameters: vars.to.regress = c("nCount_RNA", "percent.mito"), variable.features n = 2000). Any merged analysis or subsequent subsetting of cells/samples underwent the same scaling and normalization method. Cells were clustered using the original Louvain algorithm *(57)* and top 20 PCA dimensions via ‘FindNeighbors’ and ‘FindClusters’ (with parameters: resolution = 0.5) functions. The resulting merged and normalized matrix was used for the subsequent analysis.

Single Cell Type Annotation

Cell types were assigned using markers curated from several publications including Heng et al. (2019), Yao et al. (2020), Zhang et al. (2020) *(58–60)* and the Mouse cell atlas (<http://bis.zju.edu.cn/MCA/search.html>). Specific genes used to annotate broader cell types include: Mast (Tpsab1, Tpsb2, Hdc, Gata2, Kit, Hpgds , Cpa3, Tpsd1, Slc18a2 , Ms4a2, Il1rl1, Vwa5a), Adipocyte (FABP4), cDC1 (Xcr1, Itgb7, Arsb, Ckb, Fgd2, Naga, Pak1, Rab7b, Wdfy4, Ppm1m, Cd24a, Flt3, Cd83, Slamf7, Slamf8), Myofibroblast (Acta2, Tagln, Sparc, Serping1, C1r, Col1a1, Col3a1, Col6a1, Dcn, Mmp2, Ctsk, Tpsd1), B cell (Cd19, Cd74, Ralgps2, Ighm, Ms4a1, Cd19, Cd79b, Cd79a), T cell (Coxp3, Cd8b1, Cd8a, Cd4, Cd3g, Cd3e, Cd3d, Ptprc, Il7r, Saraf, Cxcr6, Nkg7, Ms4a4b, Icos, Lat, Thy1), NKT (Cd3e, Klra1, Klra6, Cd8a, Cd8b1, Ifng, Tbx21, Il2rb, Nkg7), T Naïve (Ccr7, Sell, Cd27, B3gat1, Cd28, Cd69, Fas, Il7r, Il2ra, Tcf7, Lef1), Treg (Foxp3, Il2ra, Ctla4, Ikzf2), Naïve_CM_T (Cd4, Cd8a, Sell, Klf2, Tcf7, Lef1), Rora+_Treg (Treg markers and Rora), Foxp3+Rora+_Treg (Treg markers and Foxp3, Rora, Il17, a, Il17f, Ccr6), Monocyte/Macrophage (C1qa, Csf3r, Kit, Csf1r, Cx3cr1, Itgax, Mrc1, Ly6c2, Ccr2, Ccl2, ), Macro_Vegfa (Spp1, Vegfa, Mmp12, Adam8, Cd274, Cd63, Thbs1, C3ar1, Il1rn, Clec4d, Emp1, Arg1, Ero1l, Hilpda, Hmox1, Sgk1), Macro_Ccl12 (C1qa, C1qb, C1qc, Ccl12, Sdc3, Lgmn, H2-Aa) , Basophil (Mcpt8, Gzmb, Il6), Neutrophil (S100a8, S100a9, Csf3r, Cd14, Il1b , Retn, Abtb1, Lcn2). Markers from Kopecky et al. to evaluate macrophage populations (C1qc, C1qa, Pf4, Apoe, Folr2, Cbr2, Ccl8, Cd207, Ccl12, Vsig4, Plac8, Ly6c2, Ccr2, Cytip, Fn1, S100a4, Thbs1, S100a6, Tmsb10, S100a8). Macrophage clusters were further refined using gene lists from Kopecky et al. (2022) *(16)* and were labeled using top differentially expressed genes in cases whether a more refined annotation could not be classified (ex. Cd209, vegfa, Arg1, Slc7a11).

Differential single-cell Expression Analyses

For cell-level and cluster-level differential expression, we used the 'FindMarkers' or ‘FindAllMarkers’ Seurat function as appropriate, using a minimum percent of 0.25 (parameter min.pct = 0.25) and looking only in the positive direction. The resulting differentially expressed genes (DEGs) were then filtered for adjusted p-value < 0.05. All differential expression analyses were carried out using the "SCT" assay.

Gene Set Enrichment Analysis

After performing differential gene expression analysis on neutrophil subset. Genes with an average log2 fold change greater than 1.0 within the N3 cluster were used as input gene identifiers in the Molecular Signatures Database (http://www.gsea-msigdb.org/gsea/msigdb/human/annotate.jsp). 35 genes were used in the comparison with the option of Species of input identifier set to Mouse and Hallmark gene sets were used to compute gene set overlaps.

Hallmark TNFα Signaling Via NF-κB Gene Set Score

To derive a gene set score for this pathway, we extracted genes from the gene set enrichment analysis website for this pathway (http://www.gsea-msigdb.org/). Genes with less than 10% expression across all cells in a given experiment were filtered out leaving a final gene set count of 160 genes. Finally we used Seurat’s ‘AddModuleScore’ function to annotate each cell with the resulting gene set.

*(55, 56)(57)*

**Immunofluorescence microscopy on** **Formalin-Fixed, Paraffin-Embedded Tissues**

Hearts were harvested and formalin-fixed paraffin-embedded (FFPE) tissues prepared – tissue was placed in 2% PFA overnight at 4°C, dehydrated in 70% alcohol, and then in paraffin. Antibodies used in IF are listed in **Table S5**. The IF protocol is listed in **Supplemental Methods**. Images were taken on a Zeiss Imager M2 Fluorescence Microscope. Quantification of the integral optical density (IOD) of staining was measured by average pixel intensity for each image using ImageJ (National Institutes of Health, Bethesda, MD).

**Immunofluorescence protocol**

Deparaffinize and rehydrate tissue in Xylene, 100%, 95% and 70% Ethanol for 10 minutes each. Antigen retrieval with citrate-based buffer by boiling for 10 minutes and cooling down for 30 minutes. Blocking endogenous peroxidase activity with 3% hydrogen peroxide in PBS for 15 minutes. For intracellular staining, permeabilize tissue with 0.1% triton X-100 in PBS for 10 minutes. Blocking with 5% normal goat serum for 30 minutes. Incubate tissue with primary antibody in 1% BSA in PBST overnight at 4° C. Secondary antibody for 1hr in the dark at RT. Mount with anti-fade mounting media with DAPI

**Supplementary Method 1,** Gating strategy for T cells and FOXP3^+^ Subsets in Blood


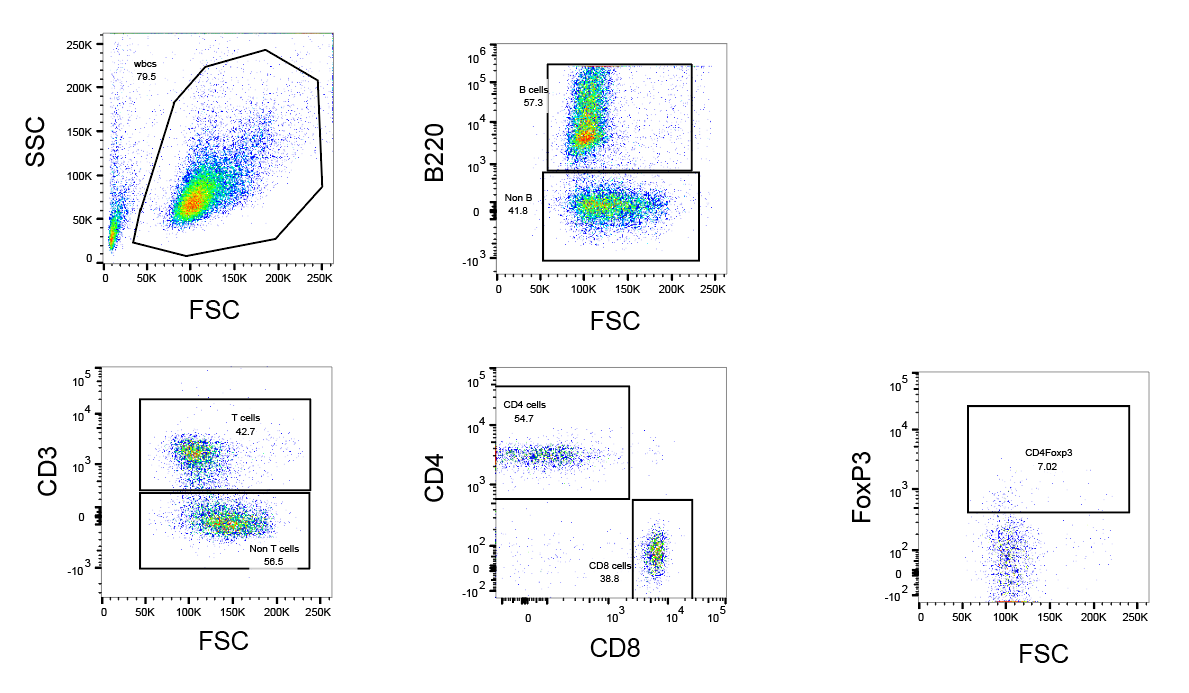


**Supplementary Method 2,** Gating strategy for CD45^+^ Hematopoietic Cells and T cells in Skin


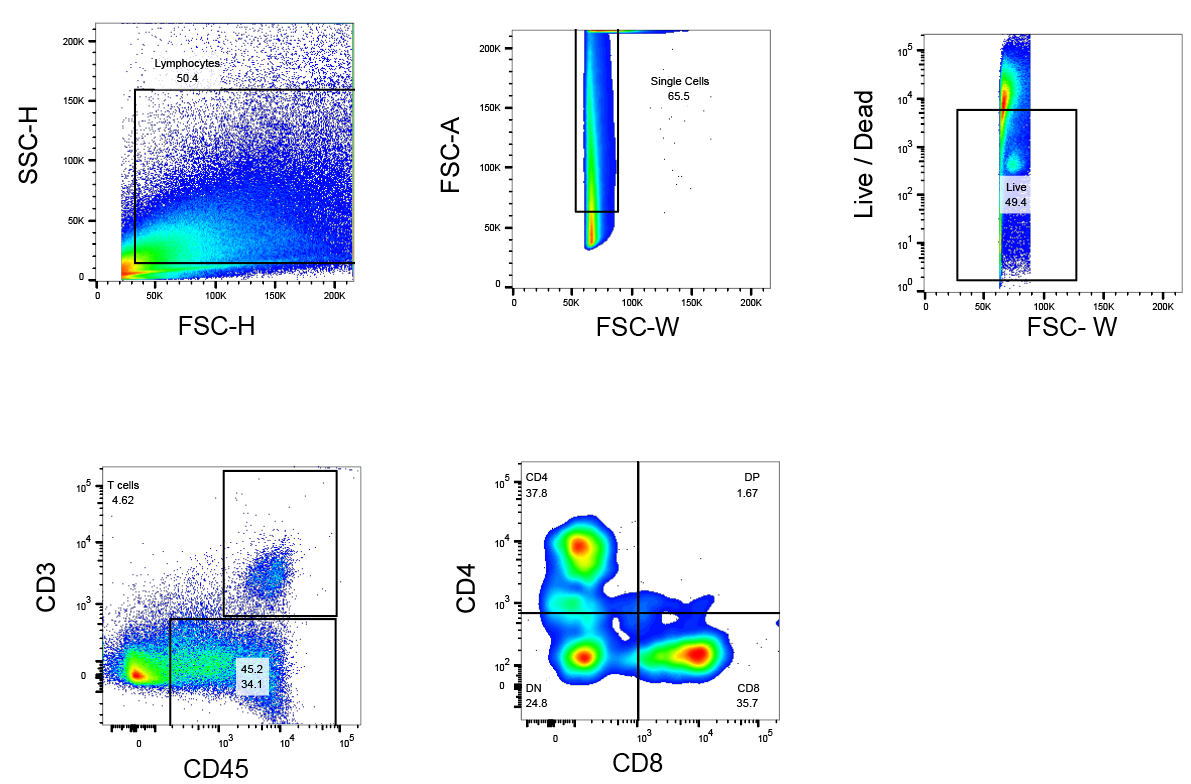


**Supplementary Method 3,** Gating strategy for T-bet^+^ T cells


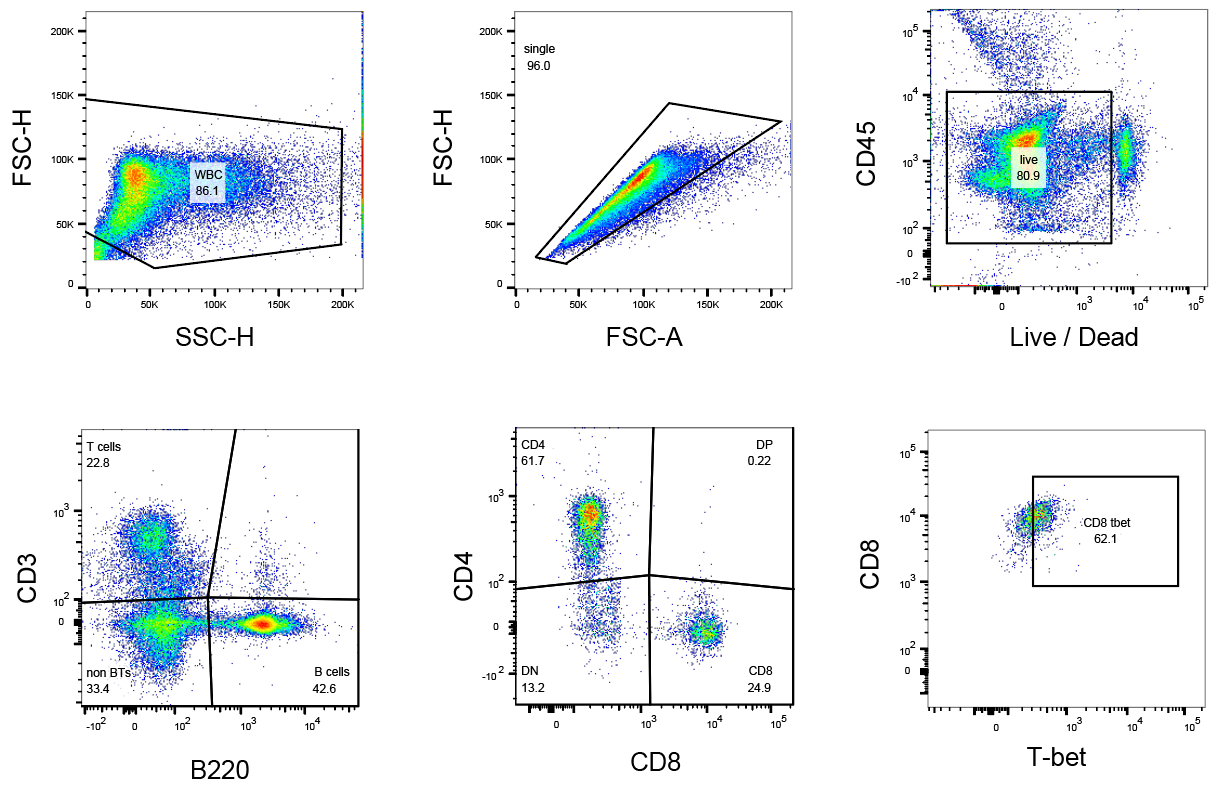


**Supplementary Method 4,** Gating strategy for T cell Subsets in Heart

**
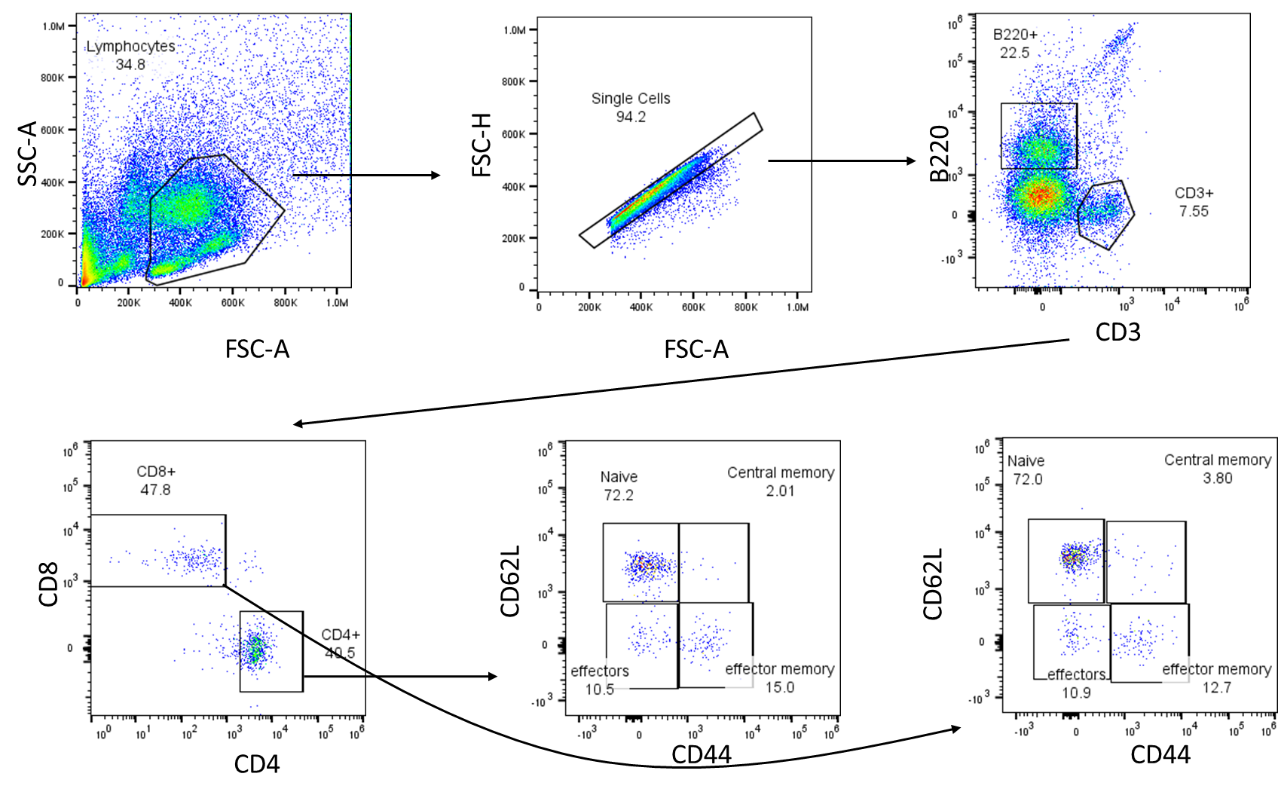
**

**Supplementary Method 5,** Gating strategy for T cell FOXP3, GATA3, T-bet, and RORγt in Heart**
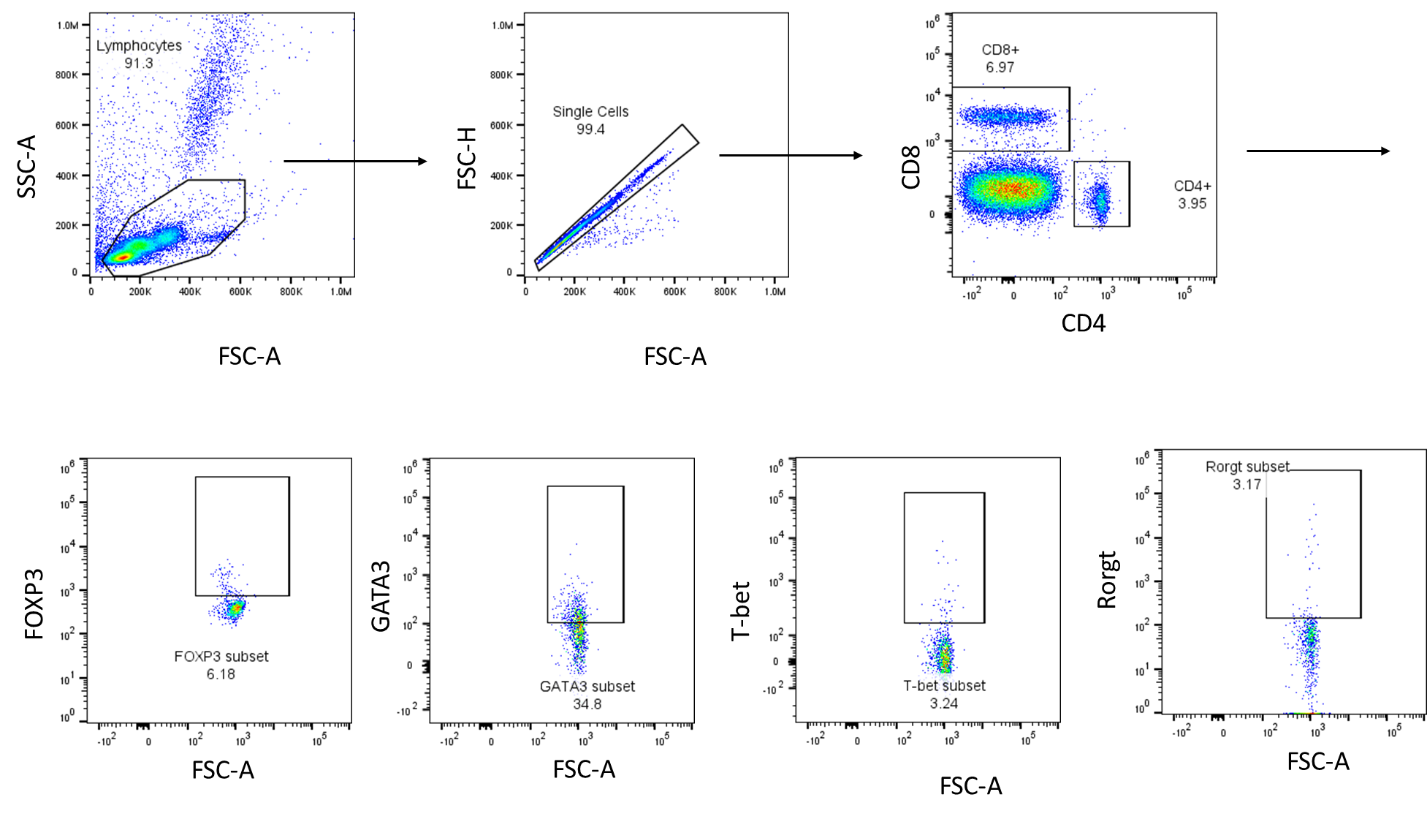
**

**Supplementary Method 6,** Gating strategy for Myeloid and T cell Recipient (CD45.1, FITC) vs. Donor Cells (CD45.2, PerCP-Cy5.5).

**
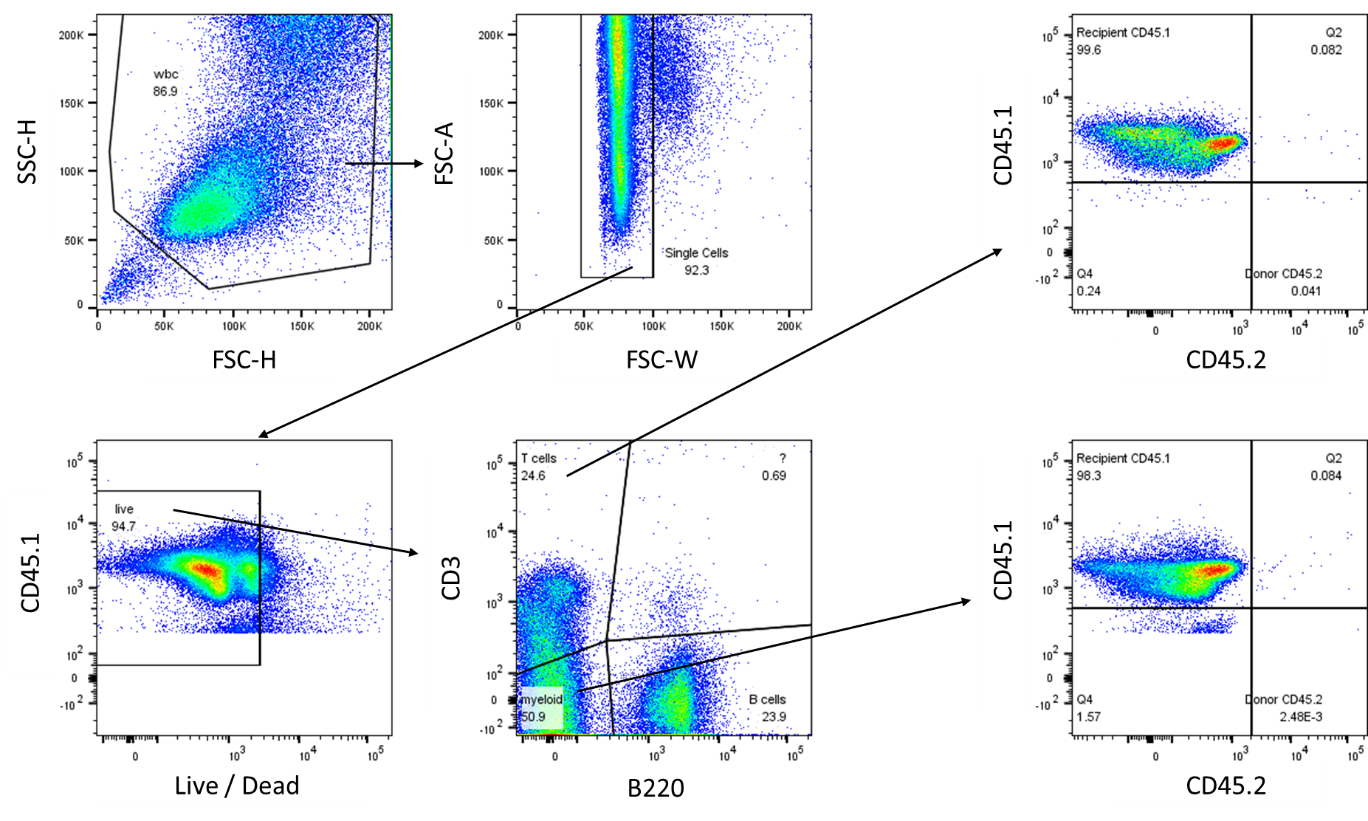
**

**Supplemental Figures:**

| Syngeneic Control | 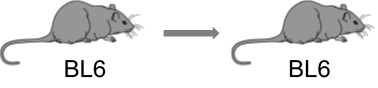 |  |
| --- | --- | --- |
| Rejection Control | 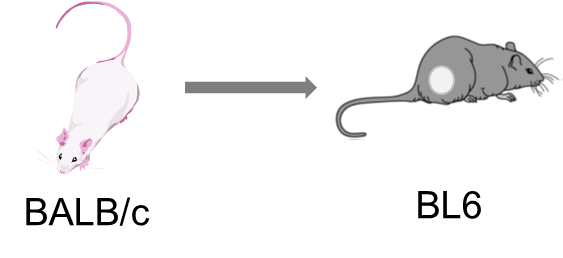 | 10% DMSO |
| Treatment Groups | 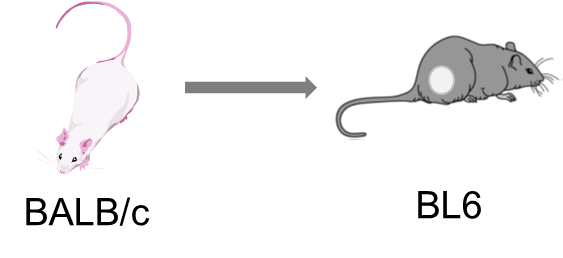 | Treatment Assignation Described In Each Experiment |
| **Fig. S1.** Fully major histocompatibility antigen mismatched BALB/c to B6 allogeneic skin grafting model. | | |

| **A**  * p < 0.05  ** p < 0.01  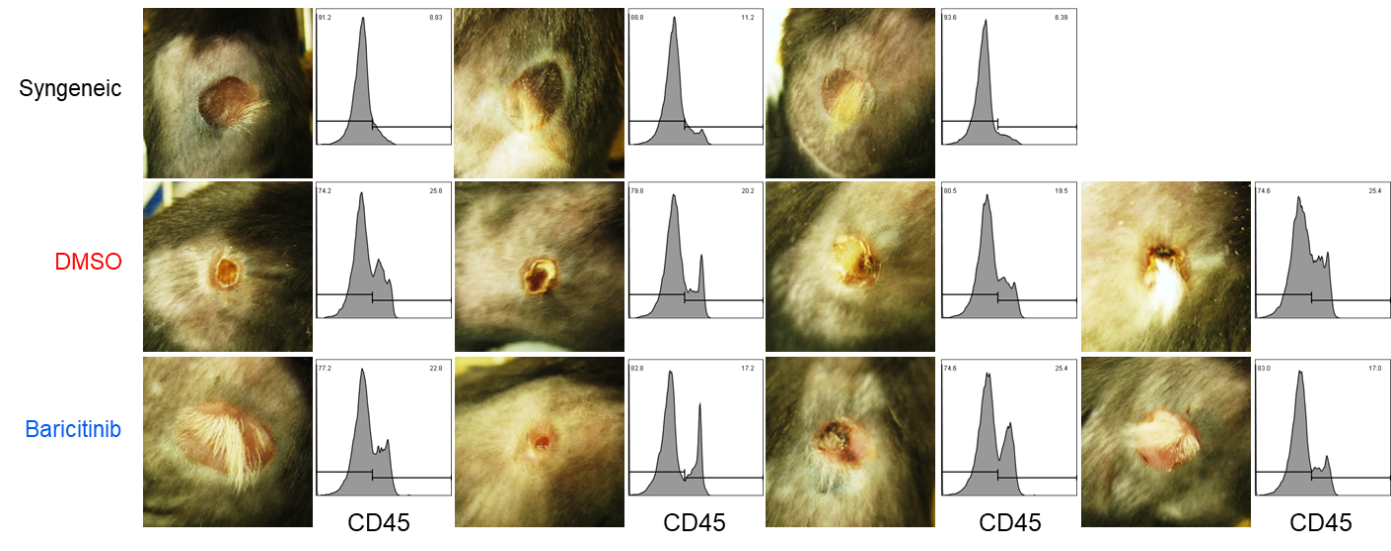 | |
| --- | --- |
| **B**   | **C**   |
| **Fig. S2. POD 14 skin graft pictures and flow cytometry in skin grafts of syngeneic, rejection control, and baricitinib treated mice.** **A.** Syngeneic grafts were healthy, rejection controls were completely rejected, and baricitinib treated mice had partially rejected grafts. **B.** CD45^+^ cells were increased in both mismatched graft groups. **C.** CD45^+^CD3^+^ T cells were increased in both mismatched graft groups. | |

|   *** p < 0.001  **** p < 0.0001 |
| --- |
| **Fig. S3.** Treatment with ruxolitinib does not increase survival of allogeneic skin grafts. Baricitinib treatment again extends the survival of grafts (p < 0.0001). |

* p < 0.05

** p < 0.01

*** p < 0.001

**** p < 0.0001

| **A. Blood PBMCs**     |
| --- |
| **B. Blood PBMCs**   |
| **Fig. S4. POD 5 flow cytometry of mononuclear cells from peripheral blood in MHC-Mismatched skin graft model. A.** Circulating WBCs, PRCs, platelets, myeloid cells, B-cells, and T cells are not different among groups. **B.** Circulating effector, memory, and naïve CD4^+^ and CD8^+^ T cells were similar among groups. |

| ****   |
| --- |
| **Fig. S5. POD 5 flow cytometry of mononuclear cells from skin in MHC-Mismatched Model.** The number of Tregs harvested from skin grafts was lower in baricitinib treated groups, compared with syngeneic controls. Otherwise, the numbers of immune cells harvested from skin grafts were similar among treatment groups. |

|  |
| --- |
| **Fig. S6. Recipient vs. Donor Cells.** Live cells from PBMCs and skin grafts groups as CD45^+^ and CD3^+^ T cell subsets. Recipient derived cells are CD45.1^+^ and donor derived cells are CD45.2^+^. In all groups, the vast majority of cells are recipient derived. |

| **A**   | **B ** | **C ** |
| --- | --- | --- |
| **D ** | **E**  **** | **F**  **** |
| **Fig. S7. T cell subsets.** Peripheral blood flow cytometry performed on POD 6 in control mice and baricitinib plus CsA treated mice, and on POD 35 seven days after withdrawl of treatment in baricitinib plus CsA treated mice. | | |

| **A** | **B**  | **C**  **** | **D** |
| --- | --- | --- | --- |
| **E**  **** | **F** | **G** **** | **H** **** |
| **Fig. S8. Mismatched heart grafts from BALB/c donors to B6 recipients, CD4^+^ and CD8^+^ T cell Markers.** Peripheral blood flow cytometry performed on POD 6 in untreated mice (vehicle) and baricitinib plus CsA treated mice, and on POD 35 (seven days after withdrawl of treatment) in baricitinib plus CsA treated mice. **(A, B)** In baricitinib plus CsA treated mice compared with vehicle, CD8^+^FOXP3^+^ regulatory T cells were reduced at POD 6. On POD 35, seven days after treatment discontinuation in baricitinib plus CsA treatmed mice, FOXP3^+^ cells increase. **(C, D)** T-bet^+^CD8^+^ T cells were significantly reduced on POD 6 in baricitinib plus CsA treated mice compared with vehicle controls. On POD 35, seven days after treatment discontinuation in baricitinib plus CsA treatmed mice, T-bet^+^CD8^+^ T cells were increased. **(E, F)**  GATA3^+^ T cells were not different bewteen groups. **(G, H)** RORγt^+^ T cells were not different bewteen groups. | | | |

**Supplemental Tables:**

**Table S1:** JAKs Inhibitory Activities of Baricitinib and Ruxolitinib

| **JAK Inhibitor** | **JAK1 IC_50_** | **JAK2 IC_50_** | **JAK3 IC_50_** | **TYK2 IC_50_** | **FLT3 IC_50_** |
| --- | --- | --- | --- | --- | --- |
| Baricitinib | 4.0 nM | 6.6 nM | 787 nM | 61 nM | - |
| Ruxolitinib | 6.4 nM | 8.8 nM | 487 nM | 30.1 nM | - |

| **Experimental Group** | **Cell Count** |
| --- | --- |
| Syngeneic Control | 1216 |
| DMSO | 2035 |
| Baricitinib | 2557 |
| CsA | 1294 |
| Baricitinib plus CsA | 1353 |

**Table S2:** Cell counts for scRNA Sequencing

**Table S3:** Flow cytometry antibodies for skin model

| **Antigen** | **Fluorochrome** | **Company** | **Catalog Number** | 𝛍**l/t-titer** |
| --- | --- | --- | --- | --- |
| live/dead | Molecular Probes | Thermo | L34962 | 0.25 |
| B220 | BUV496 | BD | 564662 | 0.75 |
| CD11b | BUV661 | BD | 565080 | 0.06 |
| GATA3 | BV421 | BD | 563349 | 0.25 |
| NKp46/CD335 | BV450 | BD | 560763 | 1.25 |
| CD8 | BV510 | BD | 563068 | 1 |
| Ly6G | BV605 | BD | 563005 | 1.25 |
| CD44 | BV650 | BD | 740455 | 0.3 |
| Ly6C | BV711 | BL | 128037 | 0.1 |
| MHC II | BV786 | BD | 742894 | 0.06 |
| CD45.1 | FITC | BD | 553775 | 0.2 |
| CD45.2 | PerCP-Cy5.5 | BD | 552950 | 0.5 |
| RORgT | PE | BD | 562607 | 1 |
| Tbet | PE-CF594 | BD | 562467 | 3 |
| FoxP3 | PE-Cy5 | Ebio | 15-5773-82 | 1.25 |
| CD11c | PE-Cy7 | eBio | 25-0114-82 | 0.1 |
| CD3 | APC | BD | 553066 | 0.75 |
| CD62L | AF700 | BD | 560517 | 0.1 |
| CD4 | APC-Cy7 | BD | 552051 | 0.06 |

**Table S4:** Flow cytometry antibodies for heart model

| **Intracellular Staining** | | | | |
| --- | --- | --- | --- | --- |
| **Antigen** | **Fluorochrome** | 𝛍**l/t-titer** | **Company** | **Catalog Number** |
| FOXP3 | FITC | 1 | eBioscience | 11-5773-82 |
| H2Kd | PerCP/cy5.5 | 1 | BioLegend | 116618 |
| CD3 | APC (I7A2) | 1 | BioLegend | 100236 |
| CD44 | APC/Cy7 | 1 | BD (Fisher Scientific) | BDB560568 |
| GATA3 | BV421 | 1 | BD biosciences | 563349 |
| CD8 | BV510 | 1 | BD biosciences | 563068 |
| CD62L | BV650 | 1 | BD (Fisher Scientific) | BDB564108 |
| CD4 | BV711 | 0.5 | BD biosciences | 563050 |
| RORγt | PE | 0.5 | Invitrogen (Fisher Scientific) | 50-245-520 |
| T-bet | PE-Cy7 | 0.5 | BioLegend | 644824 |
| **Surface Staining** | | | | |
| **Antigen** | **Fluorochrome** | 𝛍**l/t-titer** | **Company** | **Catalog Number** |
| H2Kd | FITC | 1 | BD biosciences | 553565 |
| CXCR3 (CD183) | PerCP/Cy5.5 | 1 | BioLegend | 126514 |
| CD4 | APC | 1 | BD biosciences | 553051 |
| CD49b(DX5) | APC/Cy7 | 0.5 | BioLegend | 108920 |
| CD11c | BV421 | 1 | BioLegend | 117343 |
| CD3 | BV510 | 1 | BioLegend | 100234 |
| B220 | BV650 | 0.5 | BD biosciences | 563893 |
| Ly6C | BV711 | 0.5 | BioLegend | 128037 |
| CD8 | BV786 | 1 | BD biosciences | 563332 |
| CD11b | PE | 0.5 | BioLegend | 101208 |

**Table S5:** IF antibodies

| **Antigen** | **Company** | **Catalog Number** | **Clone** |
| --- | --- | --- | --- |
| CD45 | CellSignaling | 70257 | D3F8Q |
| CD3 | CellSignaling | 78588 | E4T1B |
| CD4 | Invitrogen | MA5-15775 | 8G1B12 |
| CD8 | Novus | NBP1-49045 | 53-6.7 |
| T-bet | Invitrogen | 14-5825-82 | eBio4B10 |
| GATA3 | CellSignaling | 5852 | D13C9 |
| RORγt | Invitrogen | 14-6981-82 | B2D |
| FOXP3 | Invitrogen | 14-5773-82 | FJK-16s |
| Ly6G | CellSignaling | 87048 | E6Z1T |
| CCR2 | Novus | NBP2-35334 | 3G7 |
| Arg1 | CellSignaling | 93668 | D4E3M |
| HIF1a | Invitrogen | MA1-516 | mgc3 |
| CD68 | BioLegend | 137002 | FA-11 |
| IL6 | R&D systems | AF-406-NA | polyclonal |
| H2Kb | BioLegend | 116502 | AF6-88.5 |
